# Supplementary material for: Flowering agricultural landscapes enhance parasitoid biological control to Bemisia tabaci on tomato in south China
Source: PLoS One. 2022 Aug 2;17(8):e0272314. doi: 10.1371/journal.pone.0272314 (PMC9345349; doi:10.1371/journal.pone.0272314)
Supplement: Attachment — Submitted filename: Rebuttal letter.docx [file pone.0272314.s005.docx]

Dr Ji-Zhong Wan

Academic Editor, PLOS ONE

20 Nov 2021

Dear Dr Ji-Zhong Wan,

We are receipt of the set of comments by reviewers, and we thank you for handling of our manuscript. We thank reviewers for their valuable suggestions, each of which we have carefully considered. We anew analyzed the data and wrote our manuscript, and we added our replies after each comment separately – please find these below.

To make the results more scientific, we replicated our study in different years (2018 and 2019). In our manuscript, we have removed the original content in winter tomato fields in 2018 and added the content in summer tomato fields in 2019. To make our manuscript clear, correct, and unambiguous, after we revised our manuscript, we once again invited DBMediting for professional English language editing services. The English service company has professional English editing qualification, and the English papers edited by this company have been published more than 100 in major journals.

As a result, we revised our manuscript that we hope it is now easier to be read and comprehended. We hope you will find our revision satisfactory, and our manuscript can be accepted for publication in PLOS ONE.

Again, thank you for your editorial assistance, and we await further from you concerning our manuscript.

Sincerely yours

Dr. Xiaoming Zhang

Corresponding author

PONE-D-20-20960

Reviewers' comments:

Reviewer's Responses to Questions

1. Is the manuscript technically sound, and do the data support the conclusions?

The manuscript must describe a technically sound piece of scientific research with data that supports the conclusions. Experiments must have been conducted rigorously, with appropriate controls, replication, and sample sizes. The conclusions must be drawn appropriately based on the data presented.

Reviewer #1: Yes

Reviewer #2: Partly

**Response:** Thanks very much for your valuable comment, we agree with the comment. We want to replicate the experiment in different years to make the results more scientific. We have removed the original content in different landscape types in winter tomato fields in 2018 and added the content in summer tomato fields in 2019. In our study, through a principal component analysis (PCA), we divided the 12 sample areas into 4 kinds of landscape types, each landscape type we have set three replicates. 25 yellow sticky traps (20 cm×25 cm) and 375 monitored leaves at each sampling field were hanged and collected every 10 days until the end of the tomato growing season.

Our study wants to identify which landscape type was most favorable to protect parasitoids and enhance their effective control to *B. tabaci*. Firstly, by comparing the relative abundance of parasitic natural enemies of *Bemisia tabaci* in the same landscape, we determined that the dominant parasitic natural enemies of *B. tabaci* in different landscapes were *Encarsia formosa*. Then, by comparing the population density of *B. tabaci* and *E. formosa* in different landscapes in early, main, and late activity periods, respectively, we found that in the main activity periods of *B. tabaci* and *E. formosa*, the population density of *B. tabaci* was the highest, and the population density of *E. formosa* was the lowest in tomato planting fields in the flower landscape. Finally, by investigating the parasitism rates of *E. formosa* on *B. tabaci* nymphs in different landscapes, we found that the parasitism rates of *E. formosa* on *B. tabaci* nymphs in tomato planting fields in flower landscape was higher in the main activity period of *B. tabaci*. Therefore, we finally conclude that the flower landscape is more conducive to control the population of *B. tabaci* and enhance the parasitic effect of *E. formosa*.

2. Has the statistical analysis been performed appropriately and rigorously?

Reviewer #1: Yes

Reviewer #2: Yes

**Response:** Thanks very much, we agree with the comment.

3. Have the authors made all data underlying the findings in their manuscript fully available?

The PLOS Data policy requires authors to make all data underlying the findings described in their manuscript fully available without restriction, with rare exception (please refer to the Data Availability Statement in the manuscript PDF file). The data should be provided as part of the manuscript or its supporting information, or deposited to a public repository. For example, in addition to summary statistics, the data points behind means, medians and variance measures should be available. If there are restrictions on publicly sharing data—e.g. participant privacy or use of data from a third party—those must be specified.

Reviewer #1: Yes

Reviewer #2: Yes

**Response:** Thanks very much, we agree with the comment.

4. Is the manuscript presented in an intelligible fashion and written in standard English?

PLOS ONE does not copyedit accepted manuscripts, so the language in submitted articles must be clear, correct, and unambiguous. Any typographical or grammatical errors should be corrected at revision, so please note any specific errors here.

Reviewer #1: Yes

Reviewer #2: No

**Response:** Thanks very much. To make our manuscript clear, correct, and unambiguous, after we revised our manuscript, we invited DBMediting for professional English language editing services. The English service company has professional English editing qualification, and the English papers edited by this company have been published more than 100 in major journals. After our manuscript was rejected, we once again invited the company to edit our manuscript to make it clear and correct.

5. Review Comments to the Author

Please use the space provided to explain your answers to the questions above. You may also include additional comments for the author, including concerns about dual publication, research ethics, or publication ethics. (Please upload your review as an attachment if it exceeds 20,000 characters)

Reviewer #1: L85: “The aim of this study, therefore, was to find out which landscape type was most conducive to the colonization of parasitoids and their effective control of pests” How the manuscript can get the aim? I can’t find it in the manuscript.

**Response:** We agree with the comment, and we have revised “The aim of this study, therefore, was to find out which landscape type was most conducive to the colonization of parasitoids and their effective control of pests” to “The overall aim was to identify which landscape type was most favorable to protect parasitoids and enhance their effective control to *B. tabaci*.”. Please see lines 124-128 in revision version.

Our study wants to identify which landscape type was most favorable to protect parasitoids and enhance their effective control to *B. tabaci*. Firstly, by comparing the relative abundance of parasitic natural enemies of *Bemisia tabaci* in the same landscape, we determined that the dominant parasitic natural enemies of *B. tabaci* in different landscapes were *Encarsia formosa*. Then, by comparing the population density of *B. tabaci* and *E. formosa* in different landscapes in early, main, and late activity periods, respectively, we found that in the main activity periods of *B. tabaci* and *E. formosa*, the population density of *B. tabaci* was the highest, and the population density of *E. formosa* was the lowest in tomato planting fields in the flower landscape. Finally, by investigating the parasitism rates of *E. formosa* on *B. tabaci* nymphs in different landscapes, we found that the parasitism rates of *E. formosa* on *B. tabaci* nymphs in tomato planting fields in flower landscape was higher in the main activity period of *B. tabaci*. Therefore, we finally conclude that the flower landscape is more conducive to control the population of *B. tabaci* and enhance the parasitic effect of *E. formosa*.

L88: Method: What is mean “between 30% and 40%”, “between 15% and 20%”, “between 35% and 50%”, respectively.

**Response:** In our previous manuscript, we divided the landscape types through the proportion of the land cover types. In this version, we revised the way of divided the landscape only by proportion of the land cover types to the way of divided it by principal component analysis (PCA), to make the landscape division more scientific and the results more credible. Please see lines 150-159 in revision version.

L122-123: “Five yellow sticky traps (20 cm×25 cm) at each sampling point were then hanged in the fields” what is the distribution of the five yellow sticky traps at each sampling point?

**Response:** Thanks very much. Five yellow sticky traps (20 cm×25 cm) at each sampling point were hanged in the fields and collected every 10 days using the same five point sampling method until the end of the growing season, the five yellow sticky traps were positioned following the planting line of tomato, and the height increased with the growth of tomato. we have made relevant revisions in the first paragraph of the Sampling. Please see lines 186-187 in revision version.

L118: Sample: Which year/what time is the sample collected to analyze the population dynamics and parasitism rate?

**Response:** We have removed the original content in different landscape types in winter tomato fields in 2018 and added the content in summer tomato fields in 2019. Please see lines 159-162 in revision version.

Reviewer #2: This paper presents research results of potential value in understanding Parasitoids control effects on Whitefly. In its present form, however, it suffers from a number of problems. The authors should have someone whose first language is English revise the text. The paper’s organization and presentation of the results is sometimes confusing, making it difficult to fully appreciate the research.

**Response:** Thanks very much. To make our manuscript clear, correct, and unambiguous, after we revised our manuscript, we invited DBMediting for professional English language editing services.

Our study wants to identify which landscape type was most favorable to protect parasitoids and enhance their effective control to *B. tabaci*. Firstly, by comparing the relative abundance of parasitic natural enemies of *Bemisia tabaci* in the same landscape, we determined that the dominant parasitic natural enemies of *B. tabaci* in different landscapes were *Encarsia formosa*. Then, by comparing the population density of *B. tabaci* and *E. formosa* in different landscapes in early, main, and late activity periods, respectively, we found that in the main activity periods of *B. tabaci* and *E. formosa*, the population density of *B. tabaci* was the highest, and the population density of *E. formosa* was the lowest in tomato planting fields in the flower landscape. Finally, by investigating the parasitism rates of *E. formosa* on *B. tabaci* nymphs in different landscapes, we found that the parasitism rates of *E. formosa* on *B. tabaci* nymphs in tomato planting fields in flower landscape was higher in the main activity period of *B. tabaci*. Therefore, we finally conclude that the flower landscape is more conducive to control the population of *B. tabaci* and enhance the parasitic effect of *E. formosa*.
