# Supplementary material for: Flowering agricultural landscapes enhance parasitoid biological control to Bemisia tabaci on tomato in south China
Source: PLoS One. 2022 Aug 2;17(8):e0272314. doi: 10.1371/journal.pone.0272314 (PMC9345349; doi:10.1371/journal.pone.0272314)
Supplement: Attachment — Submitted filename: Response to Reviewers.docx [file pone.0272314.s006.docx]

Dr Yonggen Lou

Academic Editor, PLOS ONE

10 May 2022

Dear Dr Yonggen Lou,

We are receipt of the set of comments by reviewers, and we thank for the fast and efficient handling of our manuscript. They provided valuable suggestions, each of which we have carefully considered. We added our replies after each comment separately – please find these below.

As a result, we revised our manuscript that we hope it is now easier to be read and comprehended. We hope you will find our revision satisfactory, and our MS can be accepted for publication in PLOS ONE.

Again, thank you for your editorial assistance, and we await further from you concerning our manuscript.

Sincerely yours

Dr. Xiaoming Zhang

Corresponding author

Journal Requirements:

1. Please ensure that your manuscript meets PLOS ONE's style requirements, including those for file naming.

**Response:** We have revised our manuscript carefully and we ensure that our manuscript meets PLOS ONE's style requirements, including file naming.

2. Thank you for stating the following financial disclosure: Please state what role the funders took in the study. If the funders had no role, please state: "The funders had no role in study design, data collection and analysis, decision to publish, or preparation of the manuscript." If this statement is not correct you must amend it as needed. Please include this amended Role of Funder statement in your cover letter; we will change the online submission form on your behalf.

**Response:** This work was supported by Yunnan Fundamental Research Projects [grant no. 202201AT070269]; the National Natural Science Foundation of China [grant no. 31760541]; the Reserve Talent Project of Yunnan’s Young and Middle-aged Academic and Technical Leaders [grant no. 202105AC160071]; the Young Top Talents of “High-level Talents Training Support Program in Yunnan Province” [grant no. YNWRQNBJ2020291]; and the Reserve Talents Project for the 17th Batch of Kunming’s Young and Middle-aged Academic and Technical Leaders [grant no. KMRCH2019023]. One of the corresponding authors, Dr. Xiaoming Zhang, is the funder. And we have stated what role the funder took in the study. Please see lines 476-477 in revised manuscript with track changes.

3. Please amend your list of authors on the manuscript to ensure that each author is linked to an affiliation. Authors’ affiliations should reflect the institution where the work was done (if authors moved subsequently, you can also list the new affiliation stating “current affiliation:….” as necessary).

**Response:** We have amended our list of authors on the manuscript to ensure that each author is linked to an affiliation. Please see lines 5-9 in revised manuscript with track changes.

4. We note that you have referenced (ie. Bewick et al. [5]) which has currently not yet been accepted for publication. Please remove this from your References and amend this to state in the body of your manuscript: (ie “Bewick et al. [Unpublished]”) as detailed online in our guide for authors

http://journals.plos.org/plosone/s/submission-guidelines#loc-reference-style

**Response:** We have reviewed our reference list to ensure that references we have referenced has been accepted for publication.

5. We note that Figure 1 in your submission contain [map/satellite] images which may be copyrighted. All PLOS content is published under the Creative Commons Attribution License (CC BY 4.0), which means that the manuscript, images, and Supporting Information files will be freely available online, and any third party is permitted to access, download, copy, distribute, and use these materials in any way, even commercially, with proper attribution. For these reasons, we cannot publish previously copyrighted maps or satellite images created using proprietary data, such as Google software (Google Maps, Street View, and Earth). We require you to either (1) present written permission from the copyright holder to publish these figures specifically under the CC BY 4.0 license, or (2) remove the figures from your submission:

**Response:** We agree with the comment. We have removed the Fig. 1. Please see lines 96 and 138 in revised manuscript with track changes.

6. Please review your reference list to ensure that it is complete and correct. If you have cited papers that have been retracted, please include the rationale for doing so in the manuscript text, or remove these references and replace them with relevant current references. Any changes to the reference list should be mentioned in the rebuttal letter that accompanies your revised manuscript. If you need to cite a retracted article, indicate the article’s retracted status in the References list and also include a citation and full reference for the retraction notice.

**Response:** We have reviewed our reference list to ensure that it is complete and correct.

Reviewers' comments:

Reviewer #1:

**Point** 1. The word abondance should be abundance

**Response:** We agree with the comment. We have revised “abondance” to “abundance”. Please see lines 23, 155, 157, 190, 229 and Table 2 in revised manuscript with track changes.

**Point** 2. L92: add “each of” before 12 tomato field plots

**Response:** We agree with the comment. We have added “each of” before “12 tomato field plots”, Please see line 94 in revised manuscript with track changes.

**Point** 3. L320-321: This sentence is not clear to me

**Response:** We agree with the comment. We have revised this sentence to make it clearer. Please see lines 424-425 in revised manuscript with track changes.

**Point** 4. The biodiversity is one key factor influencing the landscape. The authors may have a brief description of the diversity on each of the landscape type. For example, what is the main flower in the flower field landscape? How about other landscape?

**Response:** We agree with the comment. We have written small sub-paragraph for each of the landscapes describing their diversities. Please see lines 108-129 in revised manuscript with track changes.

**Point** 5. The predatory natural enemy is always co-occurrence with parasitic natural enemy. How about the role of predatory natural enemies in the field trial sites?

**Response:** In our study, it was found that the dominant parasitic natural enemy of *Bemisia tabaci* in different agricultural landscapes was *Encarsia formosa*, which was the specific natural enemy of whitefly. Similarly, we also investigated predatory natural enemies and found that the main predatory natural enemies were. *Nesidiocoris tenuis* Reuter (Hemiptera: Miridae), *Chrysoperla sinica* Tjeder (Neuroptera: Chrysopidae), *Menochilus sexmaculata* Fabricius (Coleoptera: Coccinellidae) and *Harmonia axyridis* Pallas (Coleoptera: Coccinellidae). However, most predatory natural enemies are omnivorous, and not only control *Bemisia tabaci*, due to the abundant data, we are still conducting correlation analysis on the relevant data of predatory natural enemy insects, which will be published later.

Reviewer #2:

**Point** 1. The hypothesis is based on finding that the parasitoids of *B. tabaci* could be observed in the tomato planting fields, but the number of species and black pupae of the wasps were significantly difference under different agricultural landscapes around Kunming, Yunnan Province. However, this is not published data, and it is better to put the content in the manuscript as supplementary material.

**Response:** We agree with the comment. We have uploaded the data as supplementary data 1 (S1 Data). Please see lines 69 in revised manuscript with track changes.

**Point** 2. Some of the descriptions in the data analysis are confusing. Are the data normally distributed? The data should be transformed if it did not follow a normal distribution. Why use the LSD method instead of Tukey's HSD?

**Response:** ① We agree with the comment. We have revised the descriptions in the data analysis to make it clearer. Please see lines 193-194 in revised manuscript with track changes.

② LSD method has wider application scope than Tukey's HSD and it has high inspection efficiency. Tukey's HSD is applicable to the same number of samples in each treatment. Because the sampling number in different landscapes in the same activity period was not always the same in our study, we chose the LSD method.

**Point** 3. Some parts of the results lack the specific description of the statistical analysis results. For example, the comparison of relative abundance and population dynamics, the description of the results of specific statistical analysis is not found. Please supplement the results of statistical analysis.

**Response:** ① We have supplemented specific description of the statistical analysis results about the comparison of relative abundance. Please see lines 230-236 in revised manuscript with track changes.

② For population dynamics, we want to show the population growth and decline process of *Bemisia tabaci* and *Encarsia formosa* in different agricultural landscapes in this part, so we did not make statistical analysis. Please see lines 281-348 in revised manuscript with track changes.

**Point** 4. The description of the results is incomplete, please revise it.

**Response:** We agree with the comment. We have revised the description of the results to make it clearer and more complete. Please see lines 230-236 and 269-348 in revised manuscript with track changes.
